# Focal-HAIN: a lightweight model with adaptive modulation and hierarchical interaction for real-time crop pest and disease monitoring

**DOI:** 10.3389/fpls.2026.1778883

**Published:** 2026-03-04

**Authors:** Wei Liu, Li Xu, Xingzhi Chang, Xiaohan Long

**Affiliations:** School of Cyberspace Security, Changzhou College of Information Technology, Changzhou, China

**Keywords:** crop pest and disease detection, focal modulation, HAIN, lightweight, real-time monitoring

## Abstract

**Introduction:**

To address the problems of low detection accuracy, severe background interference, and poor real-time performance existing in current object detection models in complex agricultural monitoring scenarios, we proposed Focal-HAIN (F-HAIN), a lightweight object detection model tailored for embedded platforms.

**Methods:**

Built on the YOLOv5 architecture with design insights from RT-DETR, the proposed model incorporates two key structural enhancements to improve multi-scale feature representation and localization precision. Firstly, focus modulation was integrated into the neck network, and the F-SPPELAN module was designed to achieve adaptive and precise modulation of the feature channel based on the focus loss-guided attention mechanism. This effectively suppresses background noise and enhances the model’s response to small targets. Secondly, the HAIN module was constructed. By introducing a deep interlacing fusion strategy, feature interaction operations within the scale are embedded into the cross-scale feature aggregation path, thereby enhancing the correlation among multi-scale features and improving positioning accuracy. This study conducted comprehensive experiments on the IP102 dataset and deployed the model on a Raspberry Pi 4B embedded device for real-time performance verification.

**Results:**

The experimental results show that the mAP50 of F-HAIN can reach 90.1%. Under the same experimental conditions, compared with models such as RT-DETR, YOLOv5, YOLOv8, YOLOv10, and YOLOv11, the performance of F-HAIN on mAP50 increased by 5.5%, 6.8%, 4.9%, 5.4%, and 3.0%, respectively. Meanwhile, F-HAIN maintains a high-speed inference of 161 FPS on a high-performance workstation and was successfully deployed in an IoT-based collaborative system where a Raspberry Pi 4B serves as the edge acquisition terminal.

**Discussion:**

These findings demonstrate that F-HAIN effectively balances high detection accuracy with computational efficiency, providing a robust and deployable solution for real-time agricultural monitoring on resource-constrained edge devices.

## 1 Introduction

For modern precision agriculture, real-time, accurate detection of crop pests and diseases is a vital technical underpinning, as it directly bears on global food security and agricultural product quality (Nyawose et al., 2025). According to FAO statistics and related studies, pathogens and pests cause annual crop losses of 20%–40%, translating to billions of US dollars in direct economic damages for the agricultural sector (FAO, 2025). Currently, farmland pest and disease monitoring relies heavily on expert field surveys and farmer visual observations. These methods are labor-consuming, costly, and highly subjective, with judgments varying significantly between observers. Most critically, they perform poorly in detecting early-stage diseases or small-scale micropest outbreaks—where distinguishing visual cues are faint or absent—failing to meet the real-time monitoring demands of large-scale intensive farming. This often leads to missed optimal intervention windows and subsequent widespread pest and disease spread (Chu et al., 2023). The problem is exacerbated in on-site mobile monitoring scenarios requiring rapid responses, which is precisely the target application of the embedded platform-based detection system developed in this study.

In smart agriculture, IoT frameworks have significantly advanced context-aware decision support. For instance, A. Khan et al. proposed an IoT-assisted system for real-time soil fertility mapping to optimize fertilizer recommendations (Khan et al., 2022a). Similarly, ensemble machine learning models have been utilized by R. N. Bashir et al. for intelligent Reference Evapotranspiration (ETo) forecasting to enhance irrigation precision (Bashir et al., 2023). Furthermore, A. Khan et al. addressed saline soil reclamation by employing LSTM-based architectures to predict soil evapotranspiration (ETs) and improve the leaching process (Khan et al., 2022b). However, while these systems effectively manage abiotic factors like water and nutrients, they often lack the high-fidelity visual sensing required for early biological threat detection.

In recent years, YOLO-based target-detection algorithms have been widely adopted for crop disease and pest monitoring. Researchers have proposed various task-specific enhancements for different crops and use cases. Chen et al. improved localization of disease regions in complex backgrounds by adding an attention mechanism and a feature-pyramid enhancement strategy (Chen et al., 2022). To address detection of small or dense disease targets, Liu et al. integrated a nested residual Transformer module into the YOLOv5 model, enhancing feature extraction for tiny lesions (Li et al., 2022). Lightweight design has also increased model practicality (Li et al., 2022). Li et al. developed a lightweight YOLO-JD network for jute pest and disease identification, reducing computational cost while maintaining high accuracy (Li et al., 2022). Qi a et al. modified YOLOv5 with visual attention for tomato virus detection, improving specificity by focusing on key lesion areas (Qi et al., 2022).

At the crop-application level, different detection methods have been developed for specific crops. Soeb et al. built a tea disease detection system, YOLO-T, based on YOLOv7 for rapid identification of common leaf diseases (Soeb et al., 2023), and Bao et al. applied unmanned aerial vehicle remote sensing with an improved DDMA-YOLO model for large-scale leaf disease monitoring in tea gardens (Bao et al., 2023). For apple diseases, Zhu et al. proposed the EADD-YOLO model to identify diverse leaf diseases in complex backgrounds (Zhu et al., 2023). Gao et al. developed BAM-Net, which incorporates spatial and channel attention to segment overlapping lesions (Gao et al., 2023). Khan et al. created a real-time apple leaf disease diagnostic system to support immediate field decisions (Khan et al., 2022).

In the field of agricultural pest and disease detection, targeted optimization of YOLO-series models has become a mainstream research direction, with most studies focusing on improving detection performance for specific crops or scenarios. For instance, aiming at vulnerable crops like strawberries, Li et al. introduced the DAC module based on YOLOv4, which effectively enhanced the detection accuracy of powdery mildew and other diseases (Li et al., 2022). Similarly, Maize-YOLO achieved high-precision detection of maize pests by leveraging multi-scale feature fusion (Yang et al., 2023), while Yue et al. proposed the YOLOv7-GCA model for pepper diseases, optimizing multi-scale lesion recognition through global context attention (Yue et al., 2024). Collectively, these studies validate that scenario-specific optimizations—such as adapting to plastic-film covering or complex weed backgrounds—can significantly improve the adaptability of deep learning models in unstructured agricultural environments, laying a foundation for the deployment of detection algorithms from laboratories to embedded edge devices (Qi et al., 2023; Gao et al., 2026).

Beyond model structure optimization, advances in loss functions and practical function expansion have also promoted the development of this field. Zhang et al. proposed Focal EIoU loss function enhances the stability and convergence speed of bounding box regression, providing a theoretical basis for improving detection precision (Zhang et al., 2022). Wen et al. proposed the pest-yolo model, which further achieved the simultaneous detection and counting of multiple pests, addressing the practical needs of agricultural production (Wen et al., 2022). However, existing research still faces prominent bottlenecks, with small target representation being the most persistent challenge. In the early stages of the disease, the lesions are scarce and small similarly, early-stage pests are diminutive, and their visual features are easily obscured by complex leaf textures or crop shadows (Liu et al., 2022). This feature-scarcity issue is exacerbated in dynamic field environments with varying illumination or severe occlusion (Wang et al., 2024; Zhang et al., 2025). Notably, most current studies focus on single-crop or single-pest scenarios, lacking sufficient exploration of cross-crop generalization capabilities. Moreover, the optimization for small targets is often limited to general multi-scale fusion strategies, failing to fully account for the unique morphological and spatial distribution characteristics of agricultural small targets, which restricts the further improvement of detection performance in real-world field applications.

While the YOLO series algorithms deliver passable performance in certain application scenarios, they still fall short in structured natural environments. When it comes to small targets—such as mild crop disease symptoms—their detection accuracy can barely meet real-world agricultural requirements. In addition, in the face of complex on-site backgrounds (e.g., weed interference, fluctuating lighting conditions), the algorithms’ stability remains wanting (Jiang et al., 2023). To tackle these pain points, researchers have devised Transformer-based detection architectures, with DETR (Zhao et al., 2024) standing out as a typical representative. Building on this foundation, the real-time variant RT-DETR has been optimized to boost inference speed significantly. RT-DETR demonstrates advantages in global feature modeling and end-to-end design, offering improved handling of occluded targets. However, for embedded agricultural applications requiring real-time performance, YOLO-based architectures provide a better balance of accuracy and computational efficiency.

Based on the YOLOv5 architecture with design insights from RT-DETR’s attention mechanisms, we proposed F-HAIN, a new lightweight method for real-time crop pest and disease monitoring. F-HAIN improves small-target accuracy and robustness in complex scenes via two innovations: the F-SPPELAN module and the HAIN module. Deployed on a Raspberry Pi 4B embedded platform, the enhanced model enables efficient operation under the hardware constraints typical of grassroots agricultural applications. The aim is to meet the accuracy requirements of practical agricultural monitoring while offering a cost-effective, user-friendly surveillance solution for pest and disease detection at the grassroots.

The key contributions of this study are summarized as follows:

A novel lightweight model, F-HAIN, is proposed for accurate, real-time crop pest and disease monitoring, built upon the YOLOv5 architecture while incorporating design principles from RT-DETR.

The F-SPPELAN Module is introduced to the Neck network, integrating the Focal Modulation mechanism to achieve precise adaptive modulation of feature channels, which effectively suppresses background noise and enhances the response of small targets.

The Hierarchical Adaptive Interaction Network (HAIN) module is designed, utilizing a deep interlaced fusion strategy to integrate intra-scale feature interaction into the cross-scale feature aggregation path, which significantly improves multi-scale feature representation capacity and localization accuracy.

The F-HAIN model is deployed and validated on the Raspberry Pi 4B embedded device, demonstrating its high efficiency and practicality for low-latency monitoring.

## 2 Experimental data

### 2.1 Dataset acquisition

This study utilizes the IP102 dataset as the primary data source for experimentation. As the first large-scale benchmark dataset dedicated to crop pest detection in agricultural computer vision, IP102, a dataset specifically designed for agricultural pest detection, is extensively utilized across various research domains, including plant image recognition and pest management. The IP102 dataset, for instance, encompasses a wide range of crops such as strawberries, beans, and tomatoes, and includes various conditions like mold and leaf-spot diseases. Unlike smaller datasets, IP102’s key advantage is that all images were captured in real field environments, exhibiting complex background clutter, partial occlusions, and overlapping leaves. The dataset’s intrinsic characteristics closely replicate the real-world challenges encountered by detection algorithms in grassroots agricultural monitoring, rendering it ideal for assessing the robustness of proposed models, particularly their capacity to detect small and inconspicuous targets.

### 2.2 Dataset filtering and partitioning

**Figure 1:**
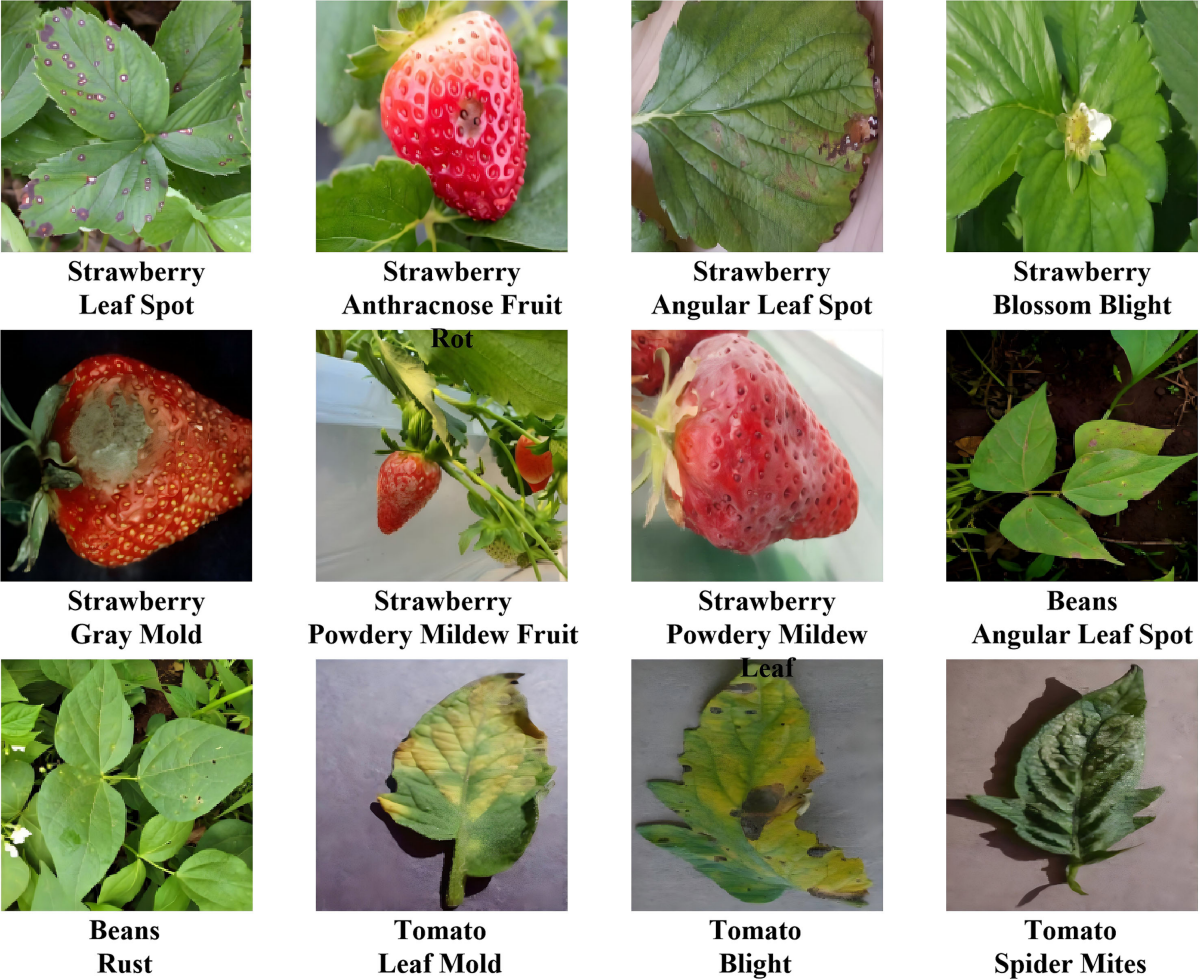
Examples of all classes of pest and disease in the dataset.

To enhance the experiment’s relevance, this study manually selected images from the IP102 dataset and chose three crops—bean, strawberry, and tomato—for evaluation. This study retained 5,483 annotated images representing 12 pest and disease types. Typical examples are shown in **Figure 1**.

The detailed distribution of samples is as follows:

Strawberry samples included 7 pest and disease categories: “Angular Leaf Spot” (541 images), “Anthracnose Fruit Rot” (194), “Blossom Blight” (356 images), “Gray Mold” (539 images), “Leaf Spot” (650 images), “Powdery Mildew Fruit” (258 images), and “Powdery Mildew Leaf” (558 images). Tomato samples included 3 categories: “Tomato Blight” (531 images), “Leaf Mold” (477 images), and”Spider Mites” (446 images). Bean samples included 2 categories: “Angular Leaf Spot” (489 images) and “Bean Rust” (414 images). The detailed images of the 12 selected pests and diseases are shown in **Table 1**.

**Table 1:** Image information of 12 selected pest and disease.

Class number	Name	Abbreviation	Number of class images
0	Strawberry Leaf Spot	S-LS	650
1	Strawberry Anthracnose Fruit Rot	S-AFR	194
2	Strawberry Angular Leaf Spot	S-ALS	541
3	Strawberry Blossom Blight	S-BB	356
4	Strawberry Gray Mold	S-GM	539
5	Strawberry Powdery Mildew Fruit	S-PMF	258
6	Strawberry Powdery Mildew Leaf	S-PML	558
7	Beans Angular Leaf Spot	B-ALS	489
8	Beans Rust	B-R	414
9	Tomato Leaf Mold	T-LM	477
10	Tomato Blight	T-B	531
11	Tomato Spider Mites	T-SM	446

To prevent bias from data partitioning and ensure reproducibility, the dataset was randomly divided into training, validation, and test sets in a 7:1:2 ratio. The training set contains 3,838 images used to learn model parameters, the validation set contains 548 images used for hyperparameter tuning and periodic performance checks during training, and the test set contains 1,097 images reserved for independent evaluation of the final model’s detection performance.

## 3 Model design and improvement

### 3.1 Basic model: YOLOv5

**Figure 2:**
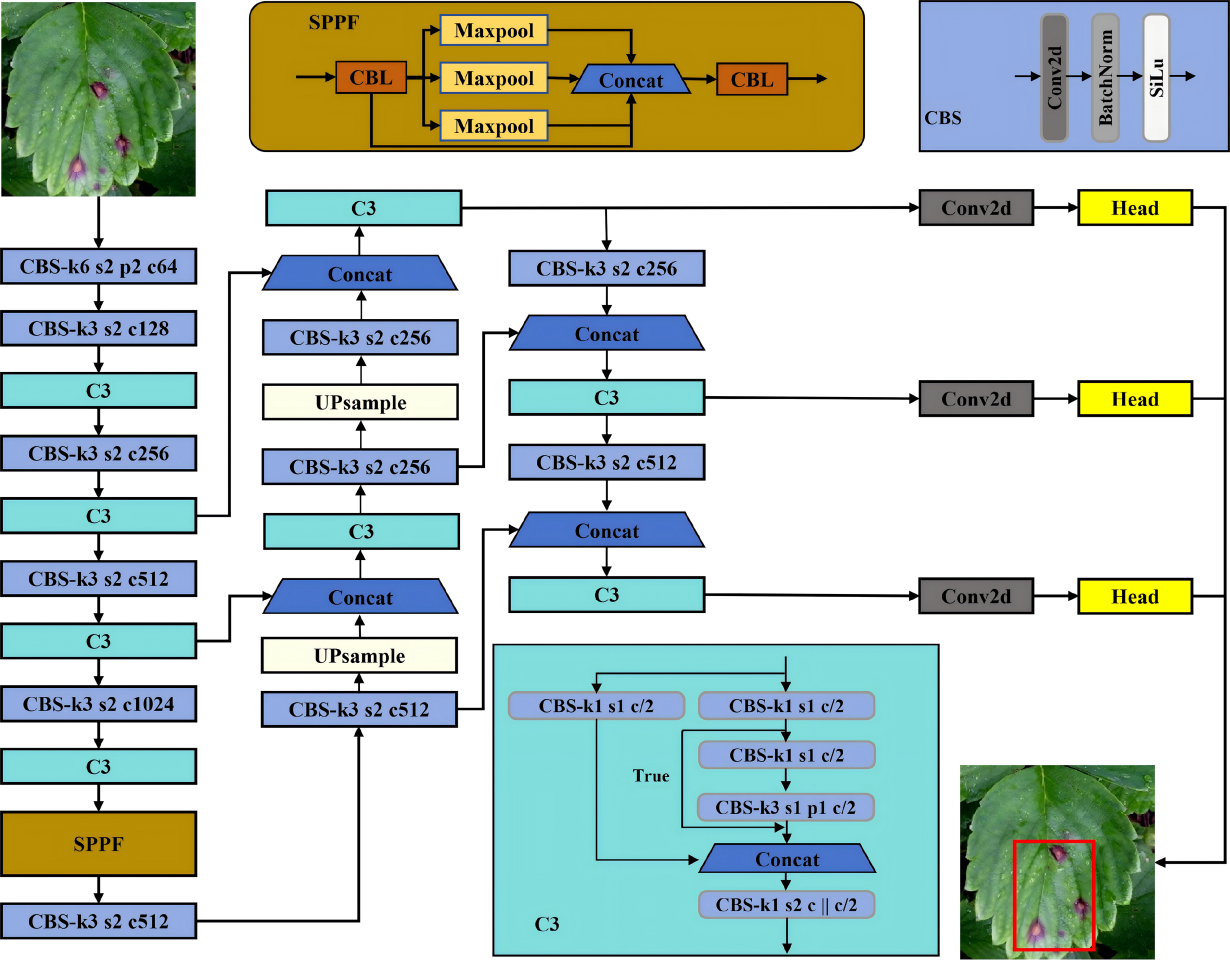
Network structure diagram of YOLOv5.

YOLOv5, a typical single-stage object detector under the YOLO family, simplifies object detection into an end-to-end regression task. This allows it to predict both the location and category of objects in one single forward pass. YOLOv5 was selected as the baseline architecture due to its proven efficiency on

embedded platforms and well-established implementation ecosystem. While RT-DETR offers advantages in certain scenarios, YOLOv5’s balance of accuracy, speed, and resource efficiency makes it more suitable for agricultural edge deployment. Such a structural design cuts down computational overhead markedly and achieves high inference speed, making it a promising option for real-time detection on resource-limited embedded platforms—well-aligned with the on-site monitoring needs of agricultural micropest detection. As illustrated in **Figure 2**, the YOLOv5 architecture comprises four core modules: input module, Backbone network, Neck network, and detection Head. 

For multi-scale feature extraction, the Backbone employs CSPDarknet53 to extract multi-scale features 156 from the input images. The input image (640×640) is downsampled via convolutional layers to yield three 157 feature maps at different scales: P3, P4, and P5. P3 is a high-resolution feature map with rich spatial detail 158 and is best suited for detecting small objects, whereas P4 and P5 have lower spatial resolution but contain 159 more abstract semantic information and are therefore better for medium and large objects.

The Neck module integrates Feature Pyramid Network (FPN) and Path Aggregation Network (PAN) to realize multi-scale feature fusion: FPN transmits high-level semantic information from top to bottom to lower layers, while PAN conveys low-level precise localization information from bottom to top. This bidirectional aggregation approach merges contextual and spatial information across different scales, thereby enhancing the detection capability for objects of various sizes. Nevertheless, in practical agricultural micropest detection scenarios, the original YOLOv5 still has certain limitations: the feature fusion in the Neck module lacks targeted enhancement for small targets, and the computational cost of the backbone network still needs optimization to adapt to long-term stable operation on embedded devices (e.g., Raspberry Pi 4B). For the final prediction, an anchor-based strategy is adopted: each feature map is preconfigured with fixed-size anchor boxes, and the network predicts bounding-box coordinate offsets, confidence scores, and class probabilities via convolution operations. Subsequently, non-maximum suppression (NMS) is used for post-processing to remove redundant detections and obtain the final results.

### 3.2 Proposed improved architecture: F-HAIN

**Figure 3:**
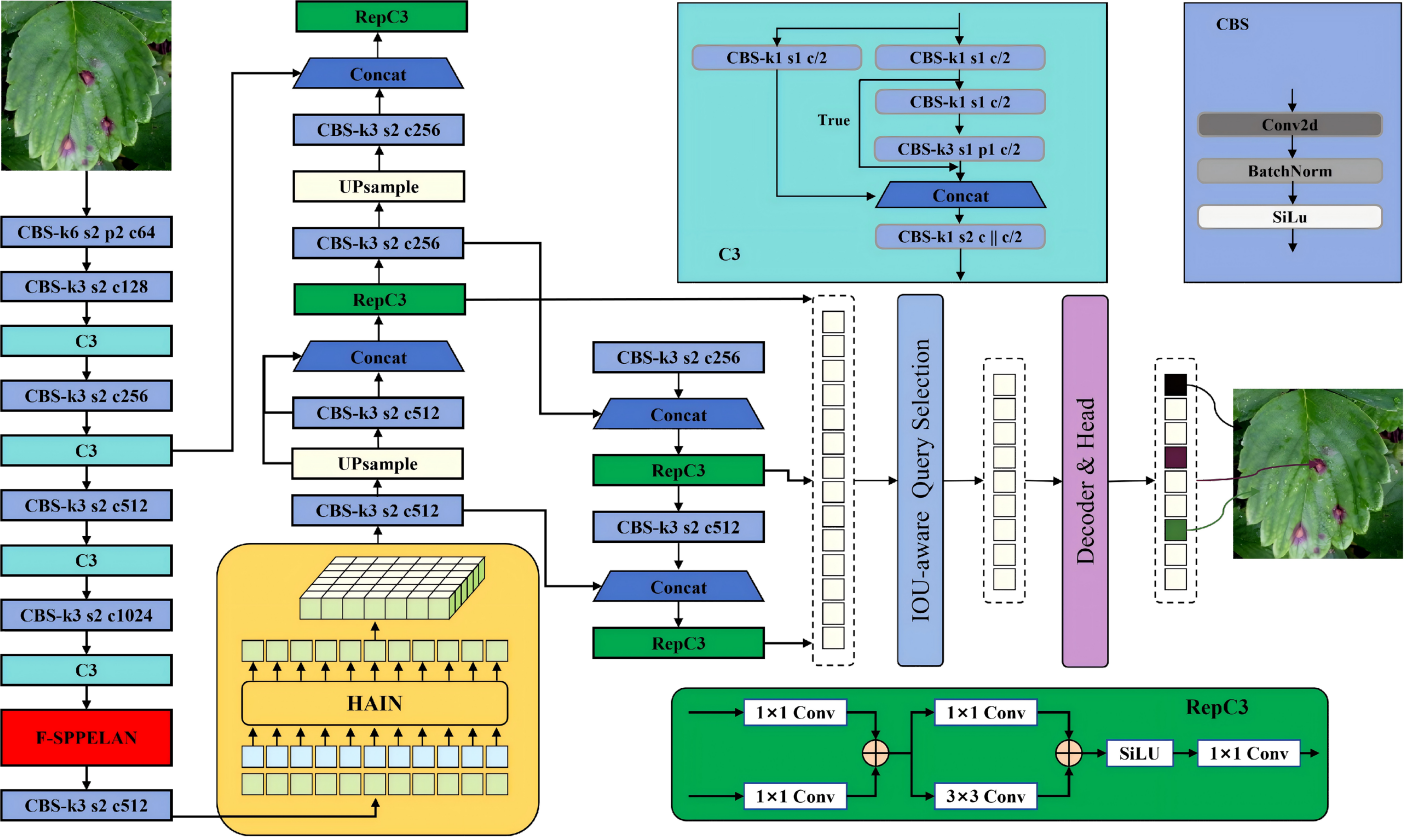
Network structure diagram of F-HAIN.

Although YOLOv5 can achieve efficient real-time detection, in actual agricultural scenarios, due to the complex background and the small size of pest and disease targets, it still encounters some challenges. To overcome these limitations and maintain computational efficiency, we proposed the F-HAIN method and made targeted improvements to it. To address this limitation, this paper proposes a novel model, F-HAIN, whose network architecture is illustrated in **Figure 3**. The model replaces the core SPPF module in YOLOv5 with a newly designed F-SPPELAN module, which enhances multi-scale target feature expression through parallel multi-scale pooling and dynamic feature weighting while preserving real-time performance. Additionally, F-HAIN incorporates the efficient hybrid encoder’s HAIN module, which processes high-level backbone features. By implementing global interaction on feature maps via a multi-head attention mechanism, the HAIN module reduces computational overhead and improves processing speed. These architectural modifications collectively mitigate critical challenges in complex scenarios, including multi-target occlusion, small target missed detection, and insufficient detection speed.

#### 3.2.1 F-SPPELAN module design

##### 3.2.1.1 Deep integration of focal modulation

Detecting crop pests and diseases is challenging because targets are small and backgrounds are complex. The SPPELAN module of YOLOV9, which extracts and enhances features across multiple scales, has difficulty balancing a global receptive field with local-detail preservation and could improve spatial feature refinement. We integrate Focal Modulation into the SPPELAN module to address these limitations. Unlike channel-attention methods that rely on global average pooling, Focal Modulation adaptively adjusts spatial weights for each pixel in the feature map. Its main advantages are: (1) extracting multi-scale context by capturing spatial information from near to far using hierarchical depthwise convolutions; (2) dynamically evaluating the contribution of different scales to each position via a gating mechanism; and (3) enhancing features of small lesions while suppressing background noise to achieve finer spatial refinement. Traditional multi-scale extraction and enhancement methods struggle to suppress redundant information and emphasize discriminative features when targets are small and backgrounds are cluttered. Although the original SPPELAN module handled multi-scale receptive fields and adaptively improved channel features, it lacked this fine-grained spatial modulation.

To address this challenge, we propose the F-SPPELAN module, which integrates focal modulation into the SPPELAN architecture. This design enables fine-grained, adaptive modulation of feature channels to amplify salient information and suppress background noise.

##### 3.2.1.2 Mathematical description of F-SPPELAN

**Figure 4:**
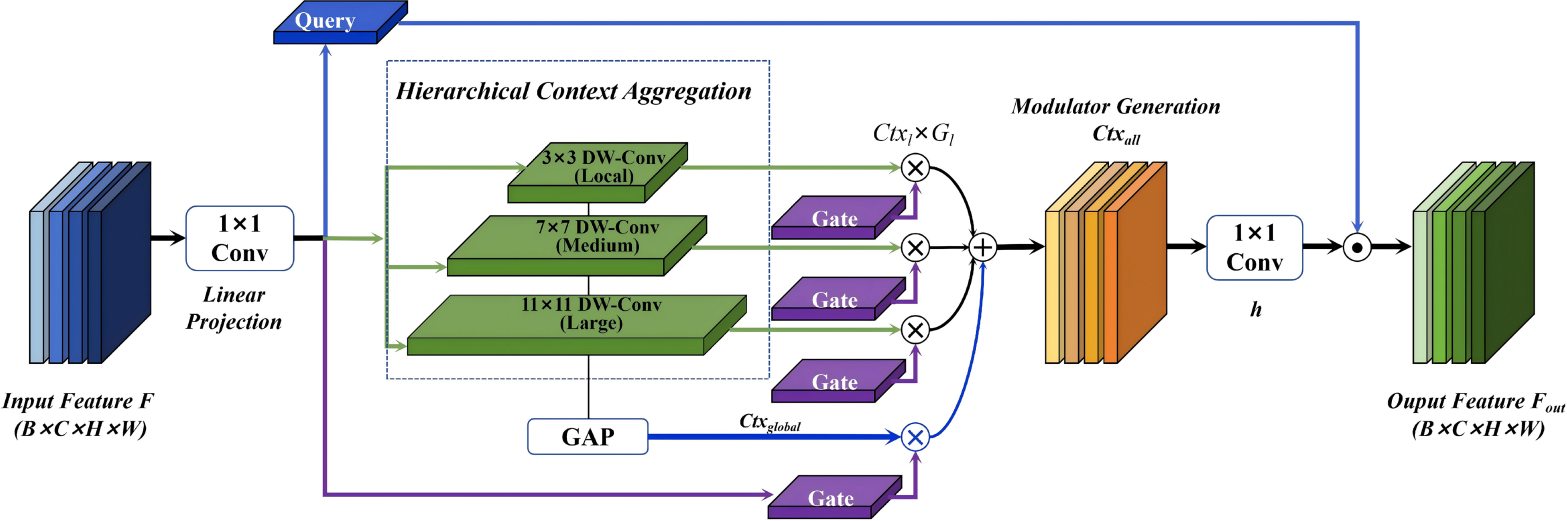
Principle diagram of the F-SPPELAN.

The F-SPPELAN implementation proceeds in three stages: linear projection, hierarchical context aggregation with gating selection, and feature modulation. The working principle diagram of the F-SPPELAN is shown in **Figure 4**.

(1) Linear projection: the input feature map $F\in {\mathbb{R}}^{B\times C\times H\times W}$ is projected by a 1×1 convolution and split into three parts: the query vector *Query(Q)*, the initial context quantity *Ctx*, and the gating signal *Gate(G)*. As shown in Equation 1.

(1) $$[Q,Ctx,G]=\text{Linear}(F),\;Q,Ctx\in {\mathbb{R}}^{C},\;G\in {\mathbb{R}}^{L+1}$$

Here, *L* denotes the set focal level.

(2) Hierarchical Aggregation and Gating: contexts at multiple scales are extracted via a sequence of depth-wise convolutions whose kernel sizes increase with level *l*. As shown in Equation 2.

(2) $$K_{l}=\text{focal}\_\text{factor}\times l+\text{focal}\_\text{window}$$

At each layer the aggregated context $Ctx_{l}$ is multiplied by its corresponding gating signal $G_{l}$for adaptive fusion. As shown in Equation 3.

(3) $$Ctx_{all}=\sum_{l=0}^{L-1}(Ctx_{l}\times G_{l})+Ctx_{global}\times G_{L}$$

The resulting $Ctx_{all}$ thus encodes multi-scale features from local to global.

(3) Feature modulation: the aggregated context is passed through another linear layer h and used as a modulator, performing element-wise (Hadamard) multiplication with the query vector *Q* to achieve adaptive feature enhancement. The final F-SPPELAN output is produced by the output projection layer. As shown in Equation 4.

(4) $$F_{out}=Q⊙h(Ctx_{all})$$

It is essential to distinguish the proposed Focal Modulation mechanism from existing attention modules and recent YOLO variants. Unlike channel-based attention such as SE or spatial-channel fusion like CBAM, which primarily perform feature re-weighting through global pooling, Focal Modulation utilizes a hierarchical gated aggregation strategy. This approach enables the model to capture multi-scale contexts and establish long-range dependencies with linear computational complexity, avoiding the quadratic overhead and loss of inductive bias associated with Transformer-based architectures. Compared to the standard CSP-based feature fusion in YOLO models, Focal Modulation provides a more refined spatial awareness, which is critical for isolating subtle pest features from complex field environments.

#### 3.2.2 HAIN module application and optimization

**Figure 5:**
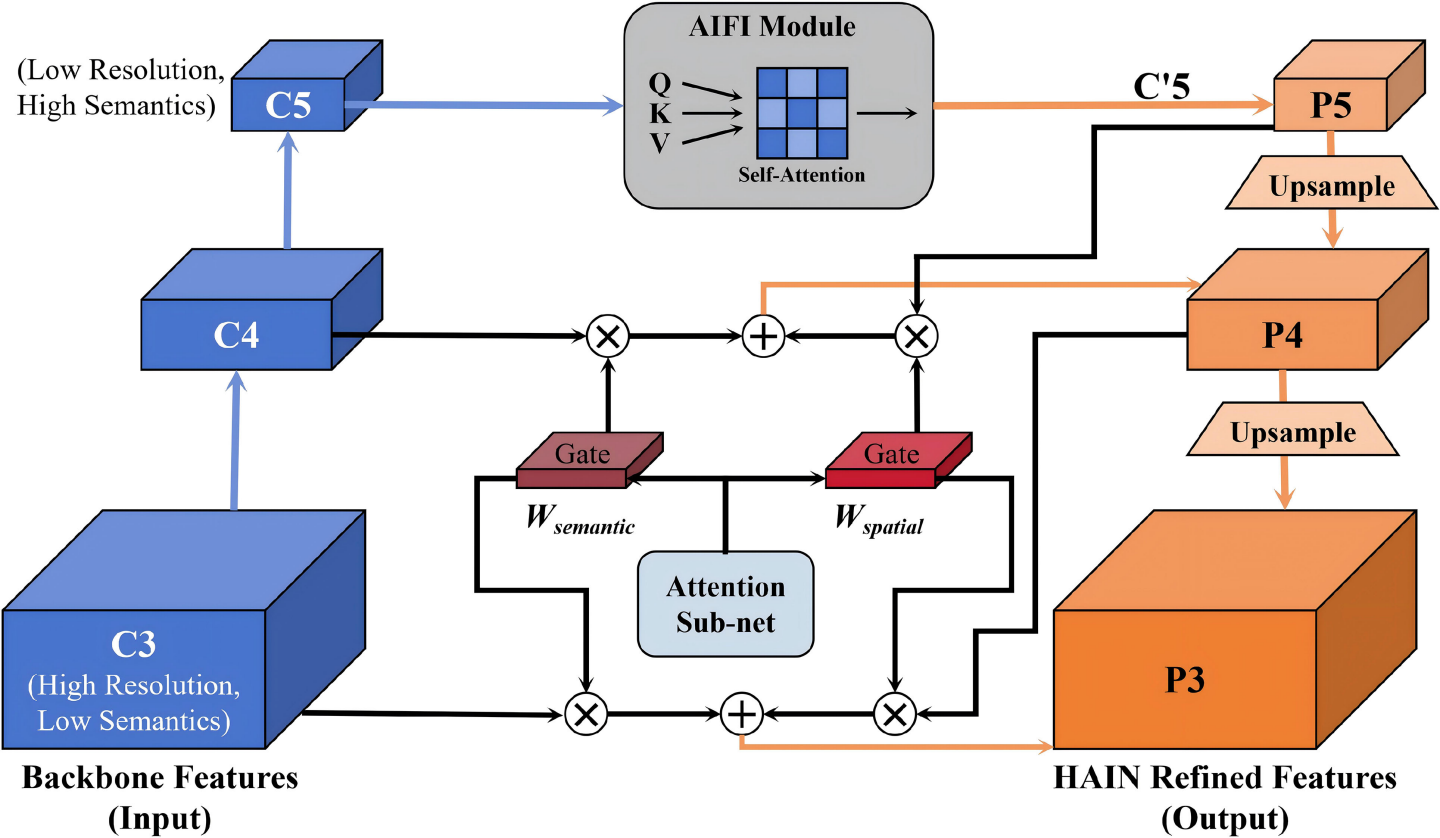
Principle diagram of the HAIN.

F-SPPELAN improves channel feature quality and small-target feature extraction, but cross-scale fusion remains limited by information inconsistency and insufficient long-range dependency modeling in the feature-pyramid Neck. To address this, we replace the FPN with the HAIN module and integrate it into the network. The HAIN working-principle diagram is shown in **Figure 5**.

HAIN’s core innovation lies in the deep interlaced fusion strategy, which embeds intra-scale adaptive interaction (AIFI) mechanisms into the hierarchical feature aggregation (HIFA) path. This ensures that the feature pyramid not only aggregates multi-scale information but also dynamically refines the information flow at every fusion step.

(1). Structure of the HAIN Module

The HAIN module is constructed as an enhanced Feature Pyramid Network (FPN), specifically designed for multi-scale feature fusion. It follows the common FPN structure of a top-down pathway combined with lateral connections, but with adaptive interaction embedded at key fusion points.

The HAIN process involves:

a) Top-down Aggregation (HIFA): High-level semantic features *P_i_*_+ 1_ are upsampled and fused with low-level features (*C_i_*) via lateral connections. This constitutes the hierarchical path.

b) Adaptive Interaction (AIFI): At each lateral connection, an AIFI module dynamically calibrates the fused features, enriching them with requisite local and global context before forwarding to the next level.

Different from standard feature pyramids, HAIN establishes a deep interlaced fusion strategy. While conventional FPN/PAN architectures focus on sequential cross-scale summation, HAIN embeds Intra-scale Adaptive Interaction (AIFI) directly into the Hierarchical Feature Aggregation (HIFA) path. This architecture ensures that semantic refinement and multi-scale aggregation occur simultaneously, mitigating the information loss typical of static weighting methods.

(2). Deep Interlaced Fusion Mechanism

HAIN achieves deep fusion by treating AIFI not as a standalone sequential block, but as a gate and refinement unit within the HIFA path.

a) Intra-Scale Interaction (AIFI) for Context Modeling: For the highest-level feature *C*5 (which has the largest receptive field but lowest spatial resolution), a simplified AIFI block based on Transformer self-attention is first applied to capture global contextual dependencies. This operation converts the global feature to $C^{\prime }5$, as shown in Equation 5.

(5) $${C^{\prime }}_{5}=AIFI(C_{5})=P_{5}$$

b) Adaptive Gated Fusion (Interlaced HIFA and AIFI): The core depth fusion occurs at the lateral connections. Instead of a simple element-wise addition, HAIN uses an Adaptive Gated Fusion (AGF) mechanism guided by AIFI principles. This mechanism dynamically weights the contribution of the high-level semantic feature ( $Up_{\left(Pi+1\right)}$) and the low-level spatial feature (*C_i_*). The refined feature *P_i_*at level *i* is calculated by Equation 6.

(6) $$P_{i}=Refine(W_{spatial}⊗C_{i}+W_{semantic}⊗Up(P_{i+1}))$$

Where:

$C_{i}$: The spatial feature from the backbone network. $Up_{\left(Pi+1\right)}$: The upsampled semantic feature from the higher layer.

Wspatial and Wsemantic: Adaptive Weight Maps generated by a lightweight attention sub-network (derived from AIFI principles), acting as dynamic gates to balance the contribution of the two features.

*Refine* (·): A lightweight local refinement convolution (e.g., 3×3 Conv) applied after the fusion.

By applying this adaptive gating at every step of the hierarchical fusion, HAIN effectively models long-range dependencies through *C*^′^5 and simultaneously ensures spatial-semantic consistency across different scales, which is critical for accurate bounding box regression and improving small target detection.

### 3.3 Comparative analysis and architectural innovation

To clarify the structural novelty of Focal-HAIN, we contrast its design rationale with incremental YOLO neck variants through two fundamental paradigm shifts tailored for agricultural monitoring.

(1) From Static Pooling to Focal-Aware Modulation: Unlike recent YOLO models (e.g., YOLOv9 to v11) that utilize static hierarchical pooling (such as SPPELAN) to expand receptive fields, our F-SPPELAN implements a focal-aware spatial modulation mechanism. While static pooling often leads to the loss of fine-grained textures in micro-pests, Focal Modulation adaptively weights spatial contexts. This allows the neck to selectively amplify pest-related features while attenuating high-frequency environmental noise like soil and leaf veins.

(2) From Sequential Fusion to Deep Interlaced Interaction: As summarized in **Table 2**, the HAIN module distinguishes itself from existing FPN/PAN variants through its structural positioning. While traditional adaptive-weighting structures primarily rely on simple learnable scalars for cross-scale summation, HAIN introduces a Deep Interlaced Fusion strategy. This strategy embeds Intra-scale Adaptive Interaction (AIFI) directly into the Hierarchical Feature Aggregation (HIFA) pathway. By doing so, it ensures that semantic refinement and multi-scale fusion occur simultaneously, effectively mitigating the semantic inconsistency often encountered when detecting micro-scale pests against chaotic agricultural backgrounds.

**Table 2:** Functional comparison between the proposed modules and existing representative methods.

Module	Reference method	Comparison of mechanisms	Solved bottleneck	Key distinction
F-SPPELAN	SPPF/ CBAM	Replaces static pooling with Focal Modulation.	Background noise and scale sensitivity.	Adaptive focal-aware spatial-channel modulation.
HAIN	FPN/PAN variants	Replaces weighted sum with Deep Interlaced Fusion (AIFI+HIFA).	Semantic inconsistency in micro-pests.	Embedded intra-scale interaction during aggregation.

## 4 Experimental analysis and performance evaluation

To ensure a rigorous and objective evaluation, we conducted all experiments under standardized conditions. To ensure the objectivity of the comparative experiments, we adopted a set of standardized hyperparameters derived from the empirical defaults of the YOLOv5 and RT-DETR frameworks. While it is acknowledged that specific architectures might benefit from bespoke tuning, our extensive pre-experiments indicated that this unified configuration allows all candidate models to reach stable convergence without significant performance bias. Specifically, we employed a warm-up strategy and a cosine annealing learning rate scheduler to mitigate the sensitivity of different architectures to the initial learning rate, thereby ensuring that the performance gains of Focal-HAIN stem from structural innovations rather than hyperparameter optimization.

Based on this unified experimental framework, we evaluated algorithm performance by comparing the detection outcomes of the baseline and improved models. The evaluation metrics employed include precision (P), recall (R), mean average precision (mAP) and F1 score. To evaluate the real-time potential, the inference speed (FPS) was measured on PC. For practical application, the model was integrated into a cloud-edge collaborative framework where the Raspberry Pi 4B captures pest images and transmits them to the server for near-instantaneous detection.

### 4.1 Experimental configuration

All training and evaluations used a single, consistent parameter set. Experiments ran on an Intel(R) Core(TM) i9-14900K (24 cores) with CUDA 12.6 and an NVIDIA RTX 4090 GPU (24GB VRAM), using the PyTorch 2.0.1 framework on Windows 11. Models were trained from scratch without using any pre-trained weights to ensure that performance gains were solely attributable to architectural innovations. Hyperparameters were: batch size 16, total epochs 300, and an initial learning rate of 1×10^−4^, with image 307 resolution standardized to 640×640. To enhance data diversity, we applied standard data augmentation strategies, including Mosaic, Mixup, and random horizontal flipping.

Considering the structural differences in the Neck between Focal-HAIN and the baseline models, a specialized learning rate protocol was adopted: a Warm-up strategy (first 3 epochs) was used to stabilize initial gradients, followed by a Cosine Annealing scheduler (minimum learning rate of 1 × 10^−6^ to ensure convergence. During the inference phase, we set the Non-Maximum Suppression (NMS) IoU threshold at 0.45 and the confidence threshold at 0.25. GIoU loss was applied uniformly. This standardized configuration demonstrates that the improvements in Focal-HAIN are a direct result of architectural innovations rather than hyperparameter bias.

### 4.2 Analysis of basic stage results

**Figure 6:**
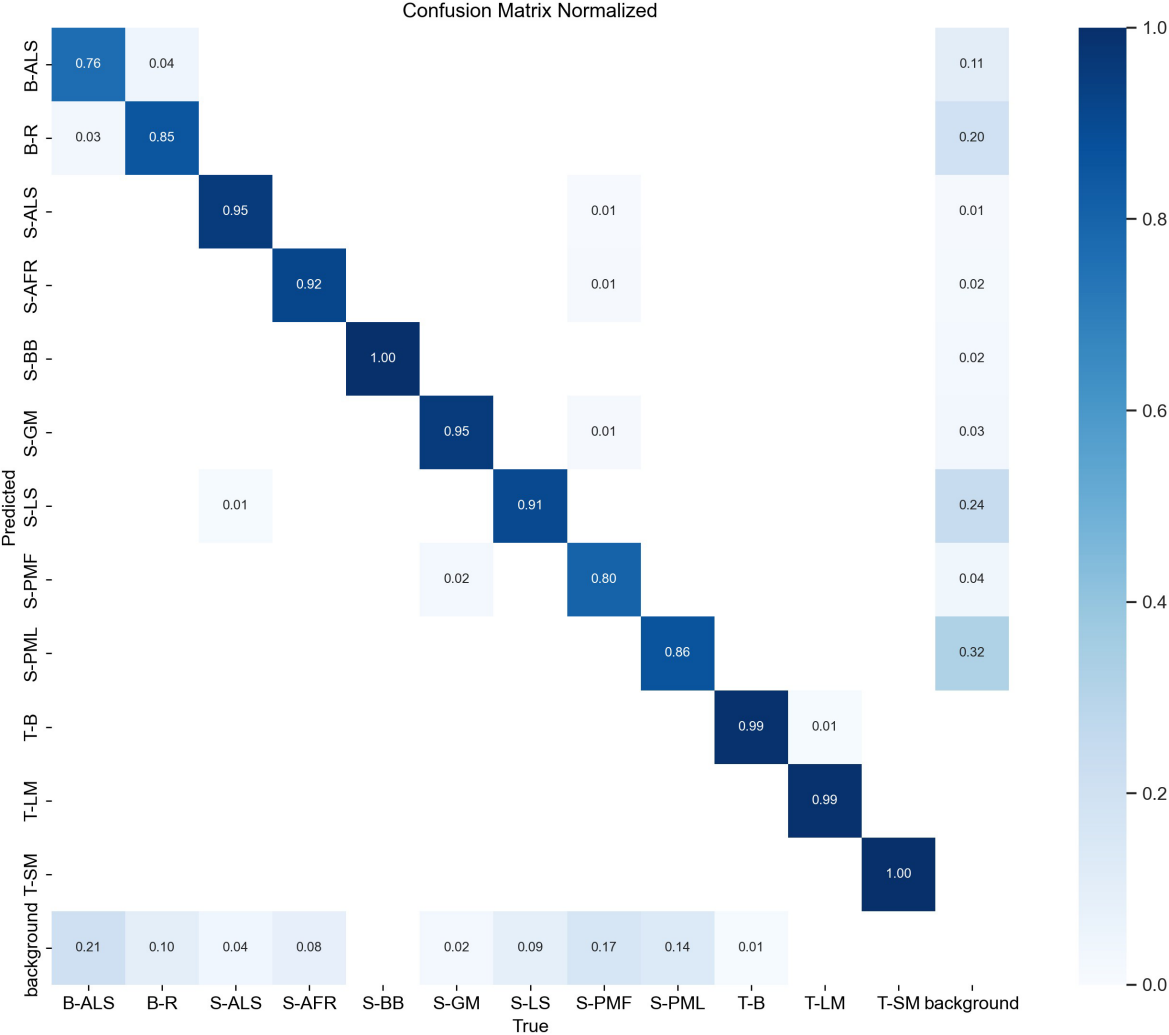
Normalized confusion matrix.

To assess model performance across categories, we visualized the predictions and computed a normalized confusion matrix. The normalized confusion matrix reports, for each category, the proportion of correct and incorrect predictions, thereby removing the effects of class imbalance and more intuitively conveying classification performance. **Figure 6** presents the model’s normalized confusion matrix on the data. Overall, the model performs well in most categories, as evidenced by the high diagonal values. However, a granular analysis of the off-diagonal elements reveals two primary failure patterns. First, inter-class misclassification is observed among visually similar diseases on the same host, such as Strawberry Leaf Spot (S-LS) and Strawberry Angular Leaf Spot (S-ALS), due to their overlapping color distributions and lesion morphologies. Second, background confusion is most noticeable in Beans Angular Leaf Spot (B-ALS) and Tomato Spider Mites (T-SM). For B-ALS, the sharp geometric edges of the lesions are occasionally confused with fragmented ground textures or soil shadows in the background. In the case of T-SM, the micro-scale, granular appearance of mites against the leaf veins poses a significant challenge for distinguishing target features from high-frequency background noise.

**Figure 7:**
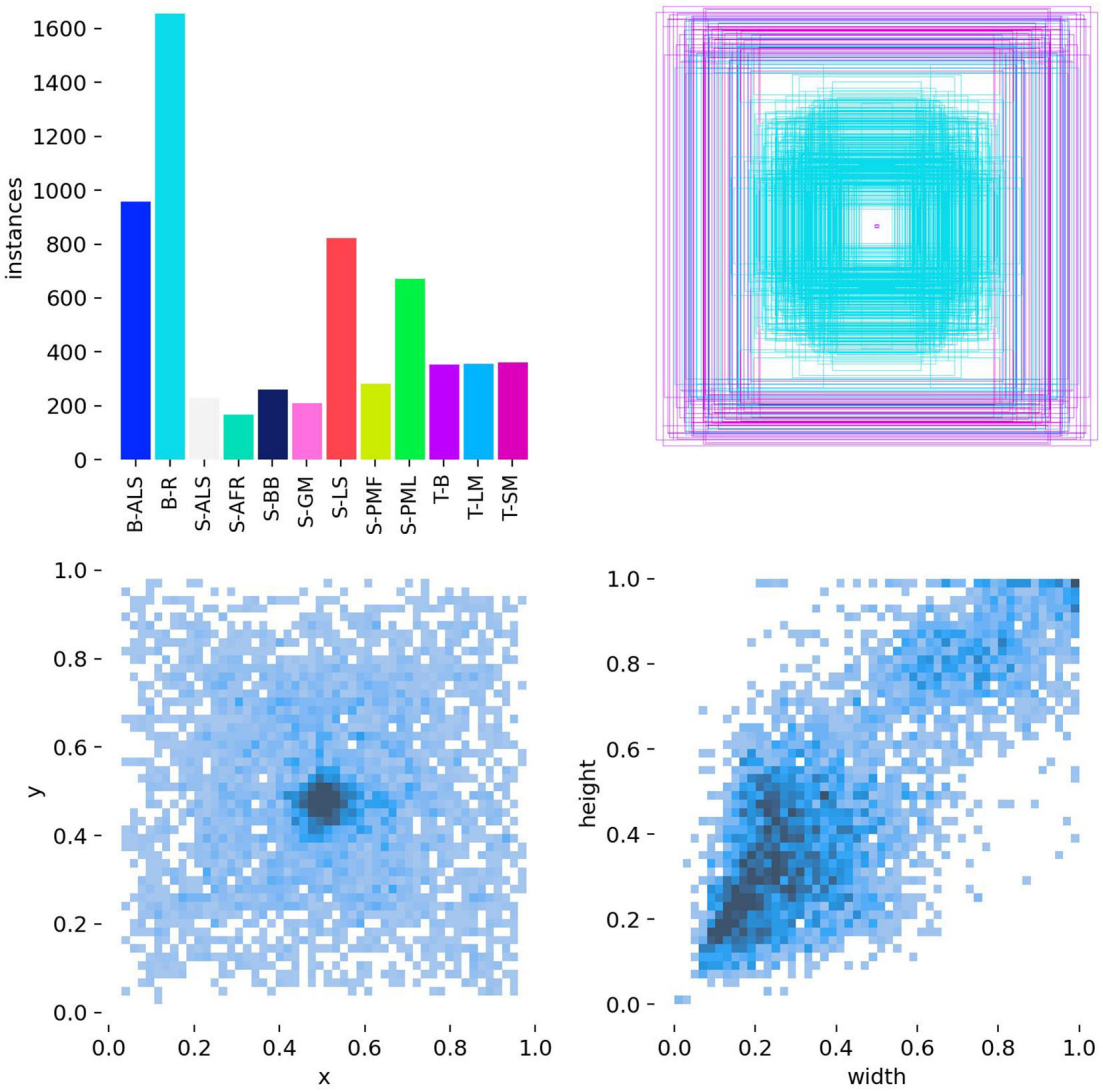
Correlogram distribution graphs of the bounding box labels.

**Figure 7** presents distributions of bounding-box labels. The top-left bar chart reports the instance count per label category, indicating the categorical distribution. The top-right plot maps bounding boxes in coordinate space: the central light-cyan cluster denotes many small, concentrated boxes, whereas the peripheral purple frames denote larger boxes, indicating hierarchical size variation. The bottom-left scatter of (x, y) coordinates shows a dense cluster near (0.4, 0.6), indicating most objects lie in the central region. The bottom-right (width, height) heatmap reveals a strong positive correlation: small widths align with small heights (dark cluster at low values) and larger widths align with larger heights, indicating bounding boxes in this dataset generally preserve relatively stable aspect ratios.

**Figure 8:**
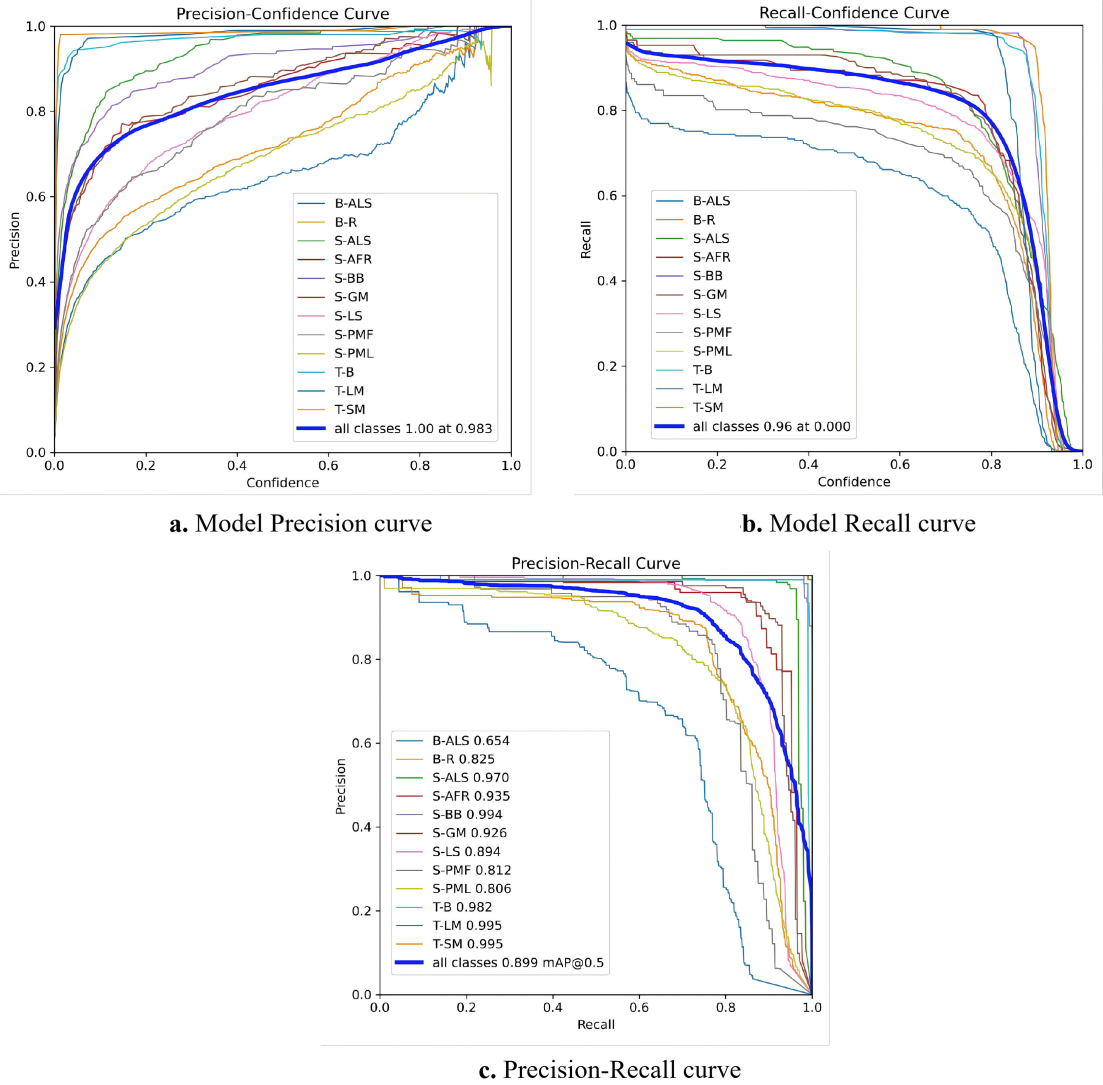
Model performance curves. **(a)** Model Precision curve. **(b)** Model Recall curve. **(c)** Precision-Recall curve.

**Figure 8** assesses model performance via three curves: precision-recall (a), precision-confidence (b), and recall-confidence (c). In **Figure 8a** classes like S-BB, T-LM and T-SM maintain around 0.9 precision across wide recall ranges, reflecting robust feature learning, while B-ALS underperforms (0.654) likely due to insufficient samples or high intra-class variability. The all classes curve yields an mAP50 of 0.899, verifying strong cross-category generalization. Linking subplots, **Figure 8b** shows precision surges with confidence and plateaus near 1.0, hitting perfect precision at a 0.983 threshold—proving high-confidence predictions are error-free. **Figure 8c** reveals a precision-recall trade-off: recall peaks at 0.96 at low confidence but drops sharply above 0.8. Collectively, these curves guide practical deployment: low thresholds maximize capture, while 0.983 filters noise for reliable detections.

**Figure 9:**
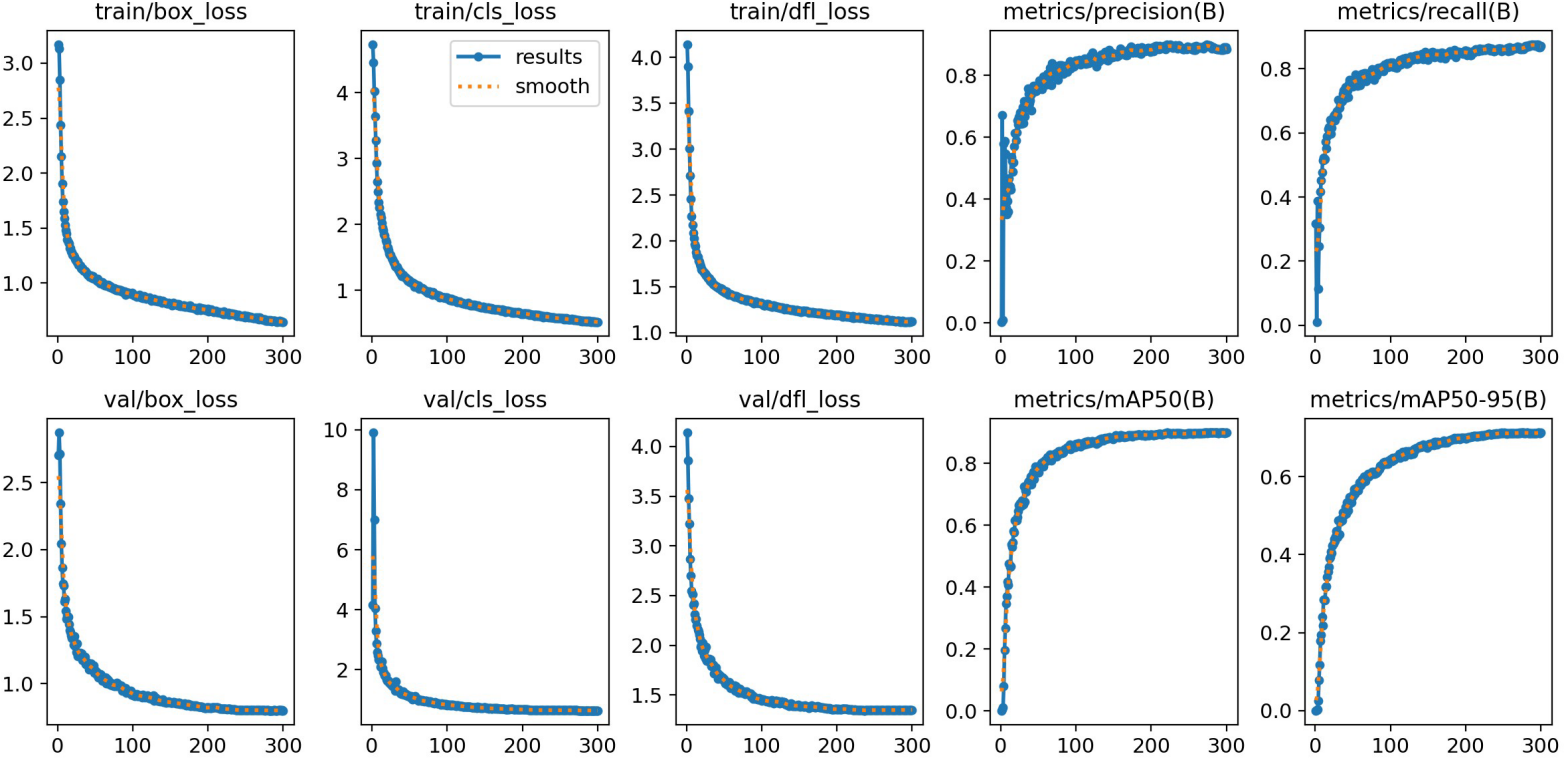
Training metrics of the F-HAIN model.

**Figure 9** depicts the 300-epoch training dynamics of the F-HAIN model, with raw (blue) and smoothed (orange) curves tracking both loss metrics and detection performance. During the first 50 epochs, all loss curves—including train/box loss, train/cls loss, train/dfl loss and validation losses—plummet sharply, then stabilize at 0.5–1.0; the tight alignment between training and validation losses clearly rules out overfitting, a key advantage for model generalization. In parallel, performance metrics show steady learning progress: metrics/precision(B) and metrics/recall(B) rise rapidly in the initial 50 epochs and plateau at 0.8-0.9, while metrics/mAP50(B) hits 0.85 by epoch 100 and peaks near 0.9. Notably, the more rigorous metrics/mAP50-95(B) stabilizes at 0.6-0.7, implying the model still has room to improve on hard targets. Collectively, these trends confirm F-HAIN’s fast convergence, stable training process and robust detection capability across target difficulty levels.

### 4.3 Ablation experiment

To systematically evaluate the contribution of each proposed module, we conducted ablation experiments based on the YOLOv5 baseline model. The ablation study has followed a systematic, incremental design to isolate the contribution of each proposed module, where we have defined three experimental groups: the Baseline (original YOLOv5 model without any modifications), single-module variants (Model A: YOLOv5 integrated only with the F-SPPELAN module; Model B: YOLOv5 integrated only with the HAIN module), and the Complete model (F-HAIN, which has incorporated both F-SPPELAN and HAIN modules). This incremental design has enabled us to quantify the individual contribution of each proposed module, assess potential synergistic effects between the two modules, and maintain experimental consistency by adopting YOLOv5 as the unified baseline across all experimental groups. The specific data are shown in **Table 3**.

**Table 3:** Ablation experiment data.

Model	Configuration	Params/Mb	GFLOPs/Gb	FPS(PC)	mAP50/%	χmAP50/%
Baseline	Original YOLOv5	26.86	15.9	153.85	84.3	–
Model A	+F-SPPELAN	27.76	16.1	149.25	87.1	2.8
Model B	+HAIN	28.44	16.6	155.85	87.8	3.5
F-HAIN	+F-SPPELAN+HAIN	24.93	16.8	161.29	90.1	5.8

The ablation results systematically demonstrate the contribution of each proposed module, linking specific structural behaviors to performance gains. The original YOLOv5 baseline achieved a mAP50 of 84.3%. Model A (YOLOv5 + F-SPPELAN) incorporates the F-SPPELAN module, which raises mAP50 to 87.1% (Δ+2.8). This improvement stems from F-SPPELAN’s focal-aware spatial modulation behavior, which adaptively suppresses high-frequency environmental noise (e.g., soil and leaf textures) while amplifying pest-related spatial contexts. This targeted modulation ensures higher recall for targets in cluttered backgrounds with only a marginal GFLOPs increase (+0.2G). Model B (YOLOv5 + HAIN) introduces the HAIN module, raising mAP50 to 87.8% (Δ+3.5). The gain is attributed to HAIN’s deep interlaced interaction behavior, where Intra-scale Adaptive Interaction (AIFI) eliminates the semantic gap between feature levels during the aggregation process. While the self-attention-based Query-Key-Value (QKV) interactions increase computational complexity (+0.7G), they provide the necessary global context to resolve semantic inconsistencies in micro-pest detection. The complete Focal-HAIN model achieves a mAP50 of 90.1% (Δ+5.8). This synergy demonstrates that the model effectively balances localized focal refinement with hierarchical global interaction, maintaining high inference speed (161.29 FPS) while ensuring the precision required for embedded agricultural deployment.

To evaluate the statistical significance and stability of the F-HAIN model, we conducted six independent runs of the F-HAIN model using different random seeds under the same hardware and hyperparameter settings. The final average mAP50 of the model was 89.73%, with a standard deviation of ±0.47%. Although random factors during the training process may introduce slight fluctuations, the performance of the model remained within a high-precision range. This variance analysis confirmed that the proposed architectural improvements have extremely high robustness and training stability.

### 4.4 Comparative experiments with other models

To further verify the validity of the proposed model, various performance indicators of the model were compared with those of other models, including RT-DETR, YOLOv5, YOLOv8, YOLOv10, and YOLOv11. The specific data are shown in **Table 4**.

**Table 4:** Comparison data with other models.

Model	Precision/%	Recall/%	F1 Score	mAP50/%	mAP50:95/%
RT-DETR	91.7	88.3	0.89	84.6	71.4
YOLOv5	92.3	88.5	0.90	83.3	72.3
YOLOv8	91.3	88.7	0.89	85.2	70.7
YOLOv10	93.3	89.6	0.91	84.7	72.2
YOLOv11	93.7	88.6	0.91	87.1	70.9
F-HAIN	94.4	91.5	0.93	90.1	79.1

Compared with RT-DETR, YOLOv5, YOLOv8, YOLOv10 and YOLOv11, the mAP50 of the improved model increased by 5.3%, 6.6%, 4.7%, 5.2% and 2.8% respectively. Meanwhile, compared with these comparison models, the mAP50:95 of the improved model increased by 7.7%, 6.8%, 8.4%, 6.9% and 8.2% respectively. These results indicate that our model outperforms the compared detectors for crop pest and disease detection in natural environments.

### 4.5 Comparison of detection results

**Figure 10:**
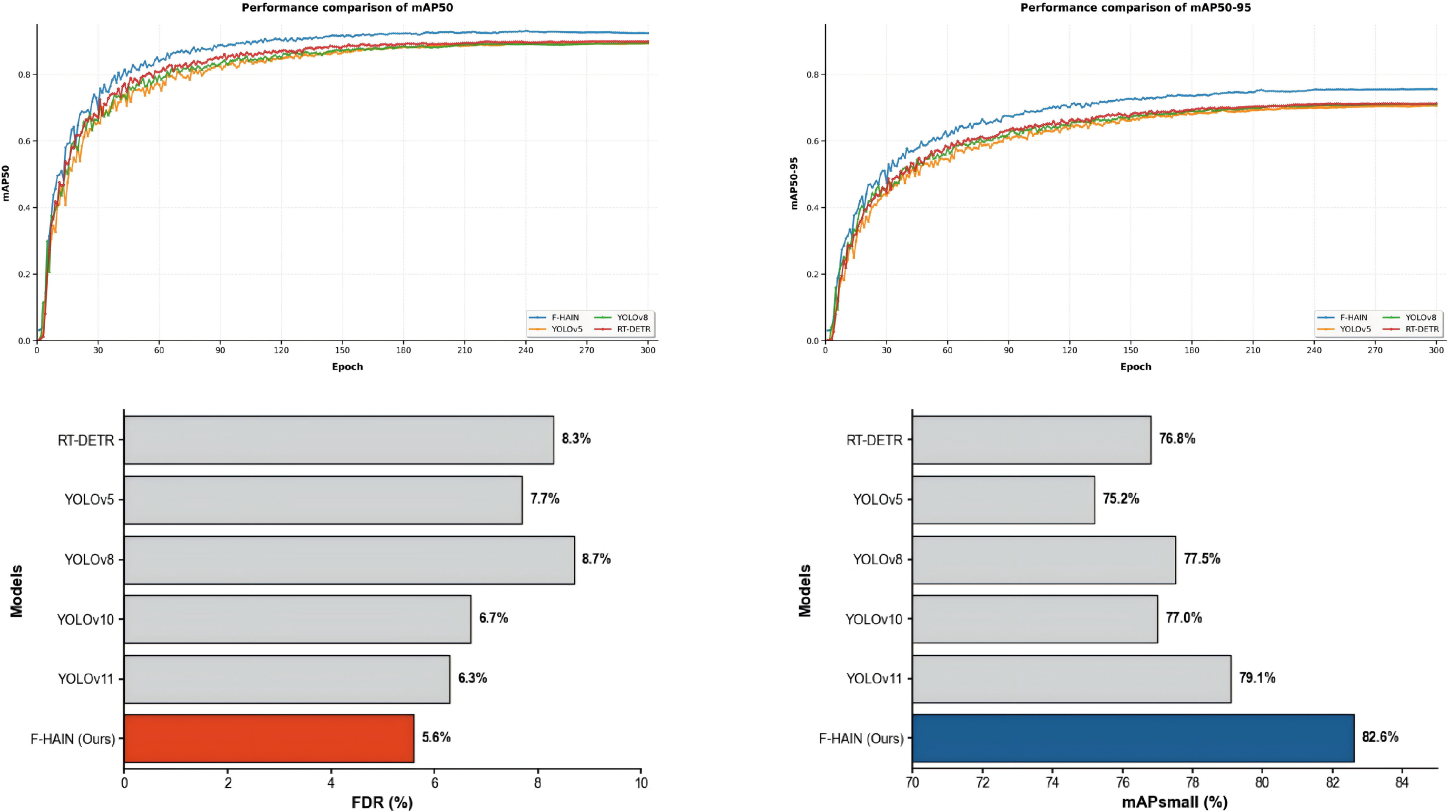
Performance comparison between the proposed F-HAIN and other SOTA models.

Controlled experiments were conducted on RT-DETR, YOLOv5, YOLOv8, YOLOv10, YOLOv11, and F-HAIN under identical datasets, training epochs, and hyperparameters to guarantee a fair comparison. Training logs were parsed to plot the comprehensive performance metrics, as illustrated in **Figure 10**.

Specifically, the two upper panels display the mAP50 and mAP50:95 curves throughout the training process, where yellow, green, red, and blue curves correspond to YOLOv5, YOLOv8, RT-DETR, and F-HAIN, respectively. To further evaluate the models under specific agricultural constraints, granular testing was performed on targeted disease subsets, as shown in the bottom panels of **Figure 10**. The lower-left bar chart presents the mAPsmall results, which were evaluated using 806 strawberry samples across five categories (S-AFR, S-BB, S-GM, S-PMF, and S-PML) where pest instances are characterized by micro-scale dimensions. The lower-right bar chart illustrates the False Discovery Rate (FDR), calculated on 518 soybean samples (B-ALS and B-R) featuring complex backgrounds such as soil and leaf interference.

Quantitative results demonstrate that F-HAIN outperforms all comparative models by notable margins across all four metrics, achieving the highest mAPsmall (82.6%) and the lowest FDR (5.6%). These performance gains are directly attributed to the HAIN and F-SPPELAN modules. Unlike conventional attention-based or Transformer-based models that often suffer from over-smoothing of fine textures, the Focal Modulation mechanism within our architecture preserves sharper boundary information through hierarchical gated aggregation. This spatial awareness allows F-HAIN to effectively enhance micro-scale feature representation for strawberry pests while suppressing non-target environmental noise in complex soybean field backgrounds, providing a more robust solution than recent YOLO variants.

To evaluate the statistical significance and stability of the Focal-HAIN model, we conducted three independent training runs using different random seeds. The model achieved a mean mAP@0.5 of 86.10% with a standard deviation of ±0.87%. Although stochastic factors in training, such as data augmentation and weight initialization, introduce minor fluctuations, the performance consistently remains within a high-accuracy range. This variance analysis confirms that the proposed architectural improvements provide robust and reproducible gains over the baseline models.

Specifically, yellow, green, red and blue curves correspond to YOLOv5, YOLOv8, RT-DETR and F-HAIN, respectively. Quantitative results demonstrate that F-HAIN outperforms all comparative models by notable margins in both metrics, with the performance gain attributed to its F-SPPELAN and HAIN modules that strengthen multi-scale feature representation and localization precision.

**Figure 11:**
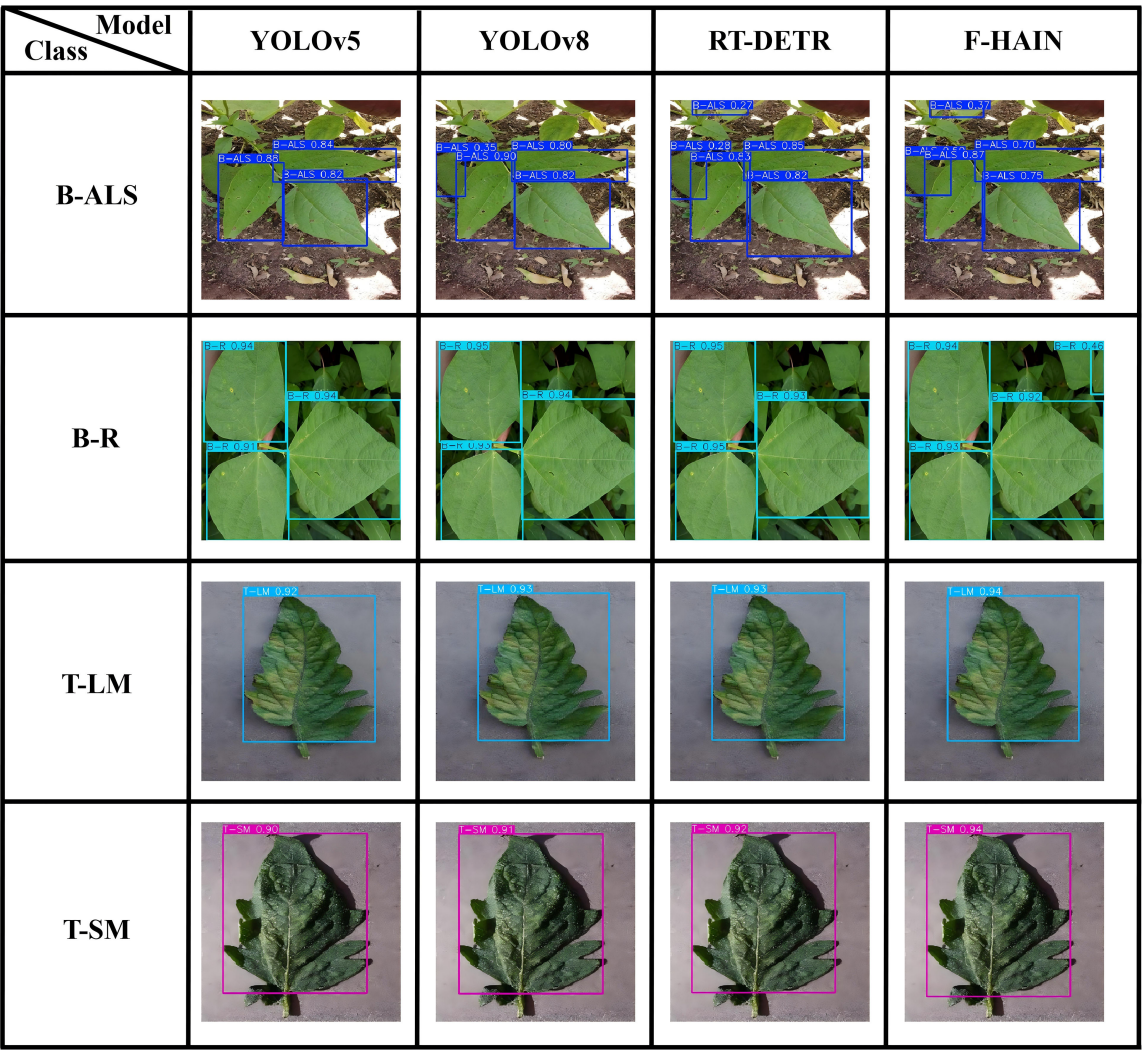
Detection results of bean and tomato.

**Figure 11** presents detection results for four pest and disease types affecting beans and tomatoes. The figure shows that F-HAIN achieves higher confidence scores than the other three models. Detection accuracy is also improved: for example, F-HAIN identified areas in B-ALS and B-R cases that the other models missed.

**Figure 12:**
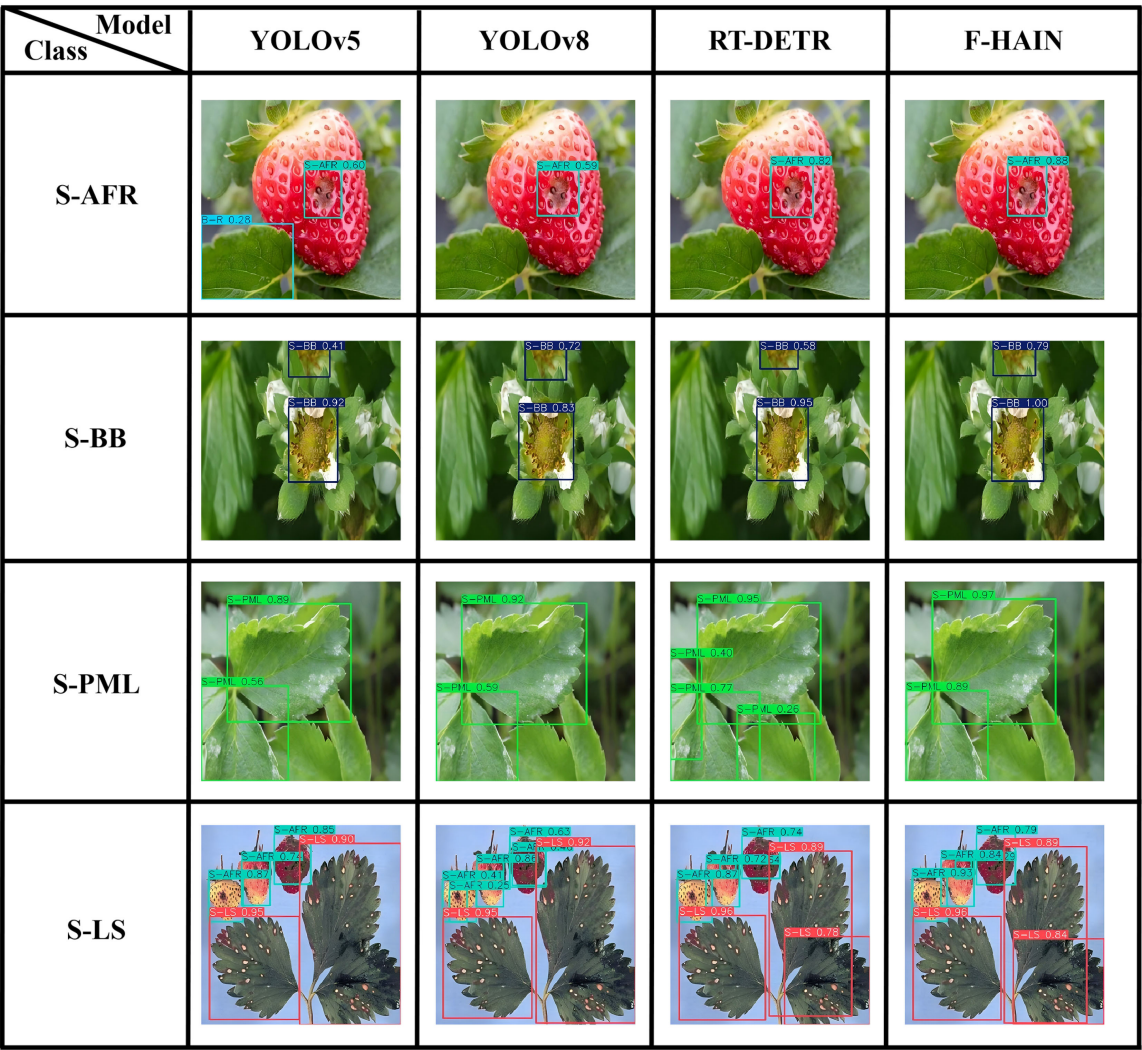
Detection results of strawberry.

**Figure 12** presents detection results for four strawberry diseases and pests. The F-HAIN detections exhibit higher confidence than those produced by the other three models. F-HAIN also yields fewer false positives for the S-LS and S-PML categories compared with the other three models.

**Figure 13:**
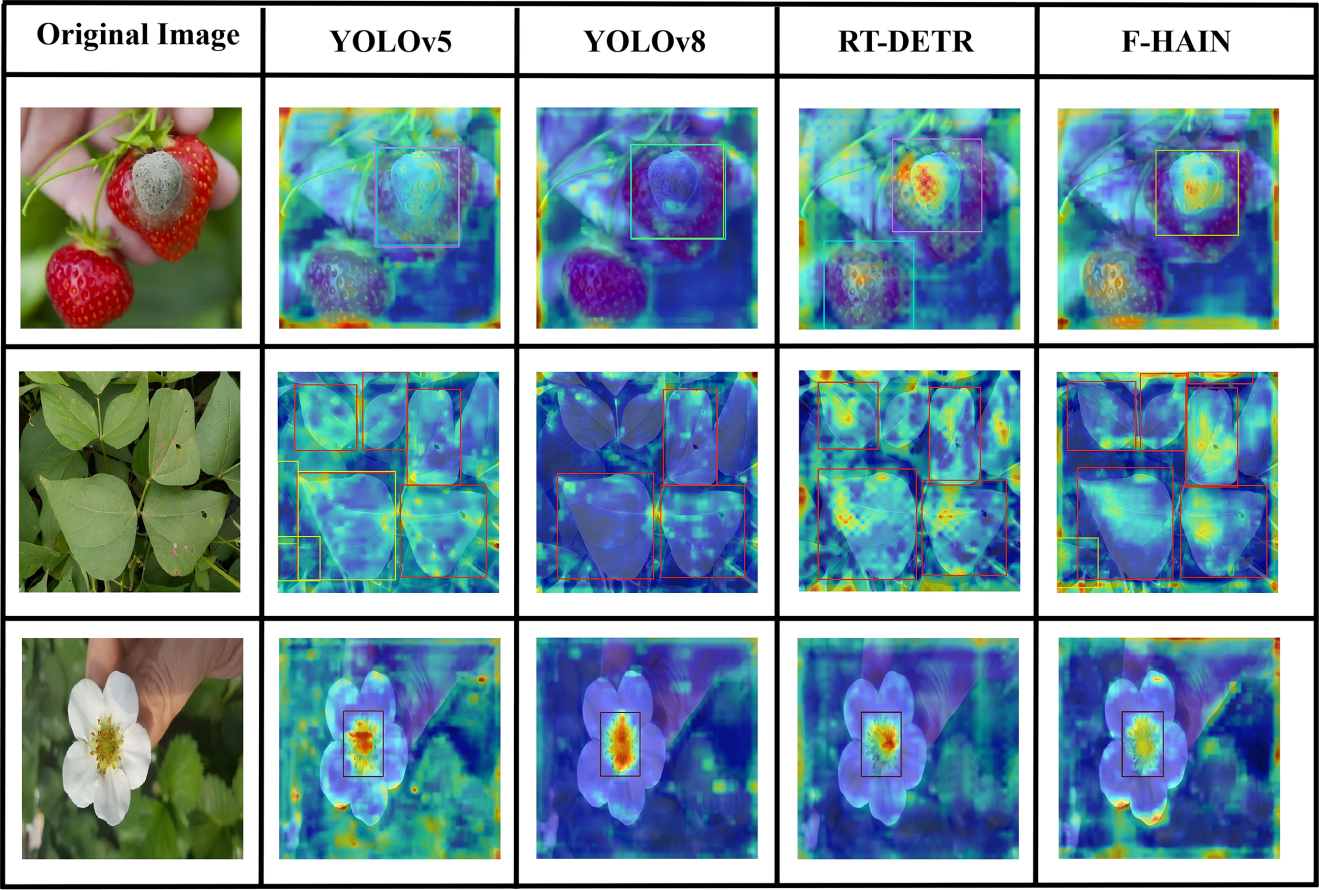
Heatmap comparison results.

In order to compare the performance differences of the three models in the object detection task more clearly, the EigenGradCAM heat map method is adopted to conduct a visual analysis of the three diseases and pests. The specific results are shown in **Figure 13**.

The heat-map graphs show that YOLOv5 and YOLOv8 produce widely dispersed response patterns, particularly near object boundaries. RT-DETR’s feature extraction within target areas is limited, which can lead to false detections; for example, in the third type there is a distinct thermal pattern beneath the petals. By contrast, the improved model yields broader high-response regions centered on key targets and shows greater robustness in edge and other non-target areas. These results indicate that the improved model captures targets more completely and thus reduces missed detections.

## 5 Experimental system based on raspberry Pi

**Figure 14:**
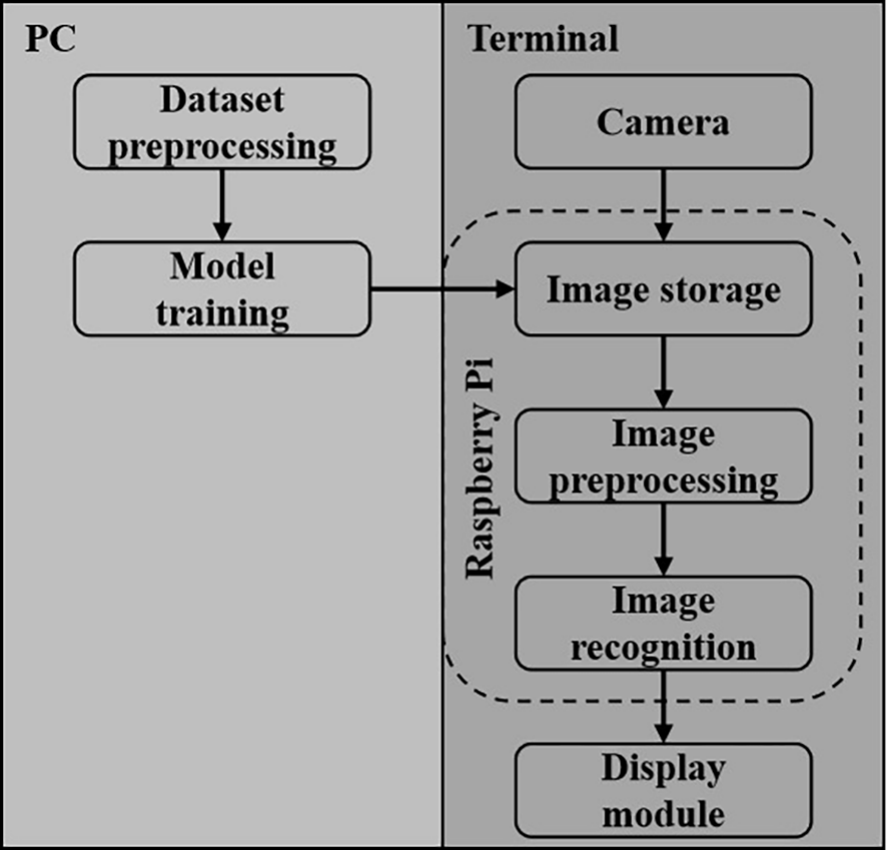
Flowchart of the Raspberry Pi detection system.

The proposed F-HAIN model was trained on a crop pests and diseases dataset and the optimized model was deployed on a Raspberry Pi 4B to evaluate detection performance and platform adaptability. Details of this deployment are shown in **Figure 14**. Experimental results demonstrate that the improved model can display detection parameters in real time on the Raspberry Pi platform and accurately detect different pests and diseases.

**Figure 15:**
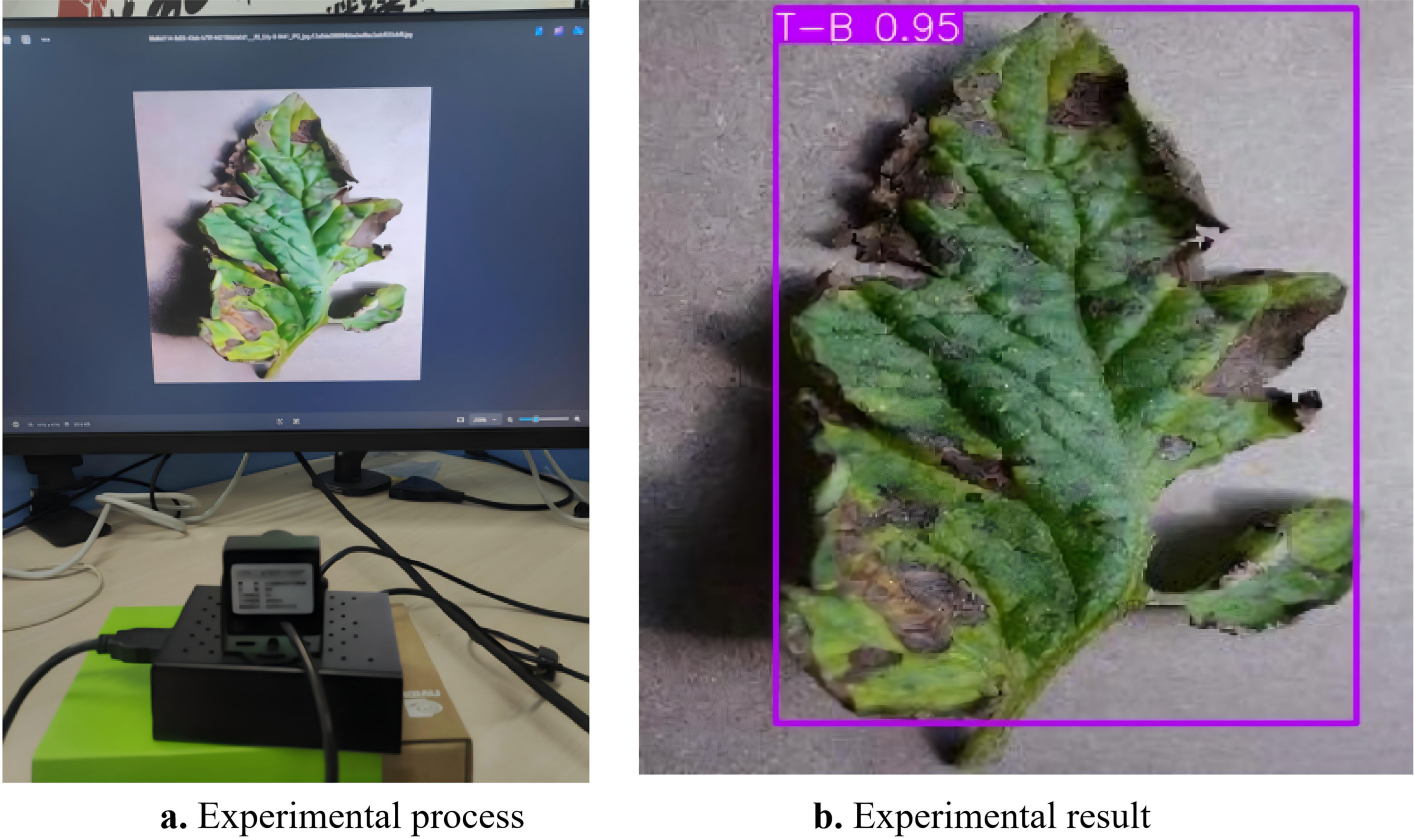
Embedded platform experiment. **(a)** Experimental process. **(b)** Experimental result.

To evaluate the F-HAIN detection model on a Raspberry Pi 4B, images were acquired with a real-time camera. On the Raspberry Pi 4B, the camera captures images, which are then processed for detection on a PC. **Figure 15a** shows the experimental setup of the embedded crop pest and disease detection system, and **Figure 15b** presents selected magnified screenshots.

The F-HAIN lightweight algorithm was tested on the portable Raspberry Pi embedded platform. The experimental results show that Raspberry PI can accurately identify pests and diseases on crops. This not only confirms the effectiveness of the algorithm in practical applications, but also provides an important reference for engineering applications.

## 6 Conclusion

In this study, we proposed Focal-HAIN, a lightweight and efficient object detection model designed for real-time crop pest and disease monitoring in complex agricultural environments. By integrating the F-SPPELAN module with Focal Modulation and the HAIN module with a deep interlaced fusion strategy, the model significantly enhances the feature representation of small targets and improves localization precision under background interference. Experimental results on the IP102 dataset demonstrate that

Focal-HAIN achieves a superior balance between accuracy and inference speed. In practical deployment, the Focal-HAIN model achieves 161 FPS on the server-side, providing sufficient throughput to support multiple Raspberry Pi 4B edge nodes simultaneously in a large-scale monitoring network. This edge to-cloud collaborative architecture ensures real-time response capabilities while maintaining low power consumption at the terminal nodes.

Despite its performance gains, the proposed method has certain limitations that warrant further investigation. Specifically, the model’s sensitivity decreases in scenarios involving low-contrast early disease spots where the lesion color closely mimics the healthy leaf tissue. In environments with highly reflective leaf surfaces, the focal modulation mechanism occasionally fails to distinguish between specular noise and small-scale pests. Furthermore, high inter-class similarity—such as different fungal diseases manifesting similar necrotic patterns on the same crop species—remains a challenge for accurate classification. Future work will focus on incorporating self-supervised pre-training to enhance fine-grained feature discrimination and exploring temporal consistency in video streams to improve the robustness of detection in dynamic, high-interference field conditions.

## Data Availability

The datasets presented in this study can be found in online repositories. The names of the repository/repositories and accession number(s) can be found in the article/supplementary material.
